# Investigating the correlations among clinical, laboratory, and imaging findings in pediatric patients with osteomyelitis and septic arthritis: a 12-year retrospective study

**DOI:** 10.1186/s12887-025-06347-4

**Published:** 2025-12-09

**Authors:** Amirali Barkhordarioon, Mahdieh Mousavi Torshizi, Ghazal Shariatpanahi

**Affiliations:** 1 Immunology, Asthma and Allergy Research Institute, Children’s Medical Center, Pediatrics Center of Excellence, Tehran University of Medical Sciences (TUMS), Tehran, Iran; 2 Department of Pediatric Rheumatology, Bahrami Children’s Hospital, Tehran University of Medical Sciences, Shahid Kiai St, Damavand Ave, Tehran, Iran; 3 Department of Pediatric Infectious Diseases, Bahrami Children’s Hospital, Tehran University of Medical Sciences, Tehran, Iran

**Keywords:** Children, Osteomyelitis, Septic arthritis, ESR, Imaging, Treatment

## Abstract

**Background:**

Septic arthritis (SA) and Osteomyelitis (OM) are critical pediatric emergencies that can lead to severe complications, including mortality. The study aimed to investigate the relationships between risk factors and clinical, laboratory, and imaging findings in these diseases.

**Methods:**

A retrospective study was conducted on 65 pediatric patients (46 diagnosed with SA and 19 with OM). Relevant demographic data, clinical, laboratory and imaging findings were analyzed.

**Results:**

45 (69.2%) patients were male. The mean age was 5.4 years for SA and 4.8 years for OM. The mean duration of hospitalization was 8.1 days for SA and 15.32 days for OM. Eight patients (12.3%) experienced specified comorbidities, leading to longer hospitalization. The most affected joints were the knee, hip, and ankle, whereas the most affected bones were the femur, tibia, and humerus. The main clinical symptoms and signs of OM included pain, tenderness, and fever, whereas those of SA included pain, limited mobility, and tenderness. Leukocytosis was observed in 57.9% of OM patients and 50% of SA patients. Erythrocyte Sedimentation Rate (ESR) elevation was found in 52.6% of OM patients and 54.3% of SA patients. Elevated C-Reactive Protein (CRP) was present in 68.4% of OM and 80.4% of SA patients. Blood cultures were positive in 23.1% of OM patients and synovial fluid cultures in 23.5% of SA patients, with *Staphylococcus aureus* being the most common organism isolated (75%). There was a correlation between hospitalization duration for SA patients and abnormal ultrasound or X-ray findings.

**Conclusions:**

Septic arthritis (SA) and osteomyelitis (OM) were more prevalent among boys aged 2–12 years. The presence of comorbidities and use of immunosuppressive medications were associated with prolonged hospital stays. Notably, fever was present in fewer than 80% of patients. Among laboratory markers, ESR was the only parameter significantly correlated with both positive culture results and longer hospitalization, emphasizing its utility in assessing infection severity and guiding follow-up. Ultrasound findings were helpful in evaluating SA severity and informing clinical management. MRI emerged as the gold standard for diagnosing OM. Finally, the low rate of culture positivity highlights the importance of initiating timely empirical antibiotic therapy, even in the absence of confirmed microbiological evidence.

## Introduction

 Osteomyelitis (OM) and Septic Arthritis (SA) are critical pediatric emergencies requiring immediate attention and intervention [1, 2]. They present significant diagnostic and therapeutic challenges, with the potential for long-term consequences such as dysfunction, length discrepancies, asymmetry, and chronic pain [3–6]. The occurrence of OM and SA ranges from 4 to 13 and 2–6 cases per 100,000 children, respectively, with higher prevalence in males [7–12].

Acute Hematogenous Osteomyelitis (AHO), the most common form of OM, typically affects the metaphysis of long bones such as the femur, tibia, and humerus and requires prolonged antimicrobial therapy, and sometimes surgical intervention [3, 8, 11, 13].

SA typically affects the hip and knee joints through monoarticular involvement [1, 7, 10, 14, 15]. The child’s SA prognosis is determined by several factors, such as age and treatment delay [2, 7, 8, 16].

Infants under 18 months display unique anatomical features, such as metaphyseal-epiphyseal blood vessels, which are closely linked to the propagation of infections between bone and joint spaces and thereby make the clinical picture more complicated and the management approach more difficult [3, 12, 15, 17].

Classic AHO symptoms (fever, pain, and reduced mobility) appear in only about half of the cases, necessitating high clinical suspicion [9, 18]. *Staphylococcus aureus* is the main causal agent of osteoarticular infections. Nonetheless, consideration should be given to other organisms in certain patient populations, such as Group B Streptococcus in infants and Kingella kingae in young children [3, 7, 9, 15]. The rising prevalence of *Methicillin-resistant staphylococcus aureus* (MRSA) complicates management and often leads to severe systemic manifestations [3, 7, 9, 15, 19]. Although plain radiography is usually the first imaging technique used, its effectiveness is limited in the early stage of infection [9, 19, 20]. MRI provides high sensitivity and specificity but is not widely used because of its high cost, limited availability, and sedation requirements [9, 15, 20].

Treatment strategies are primarily oriented toward empirical antibiotics based on local epidemiology, age, and clinical presentation [8, 12]. The heterogeneity in antibiotic resistance patterns across various geographical areas is a serious issue for the standardization of treatment [12, 19]. Previous research has shown a high resistance rate of *Staphylococcus aureus* strains to many commonly used antibiotics, such as cloxacillin, which necessitates the monitoring and prudent use of antibiotics [7]. Further orthopedic review of the condition to identify the possibility of surgical intervention, such as aspiration or debridement, is also one of the important components of the treatment plan [12].

This study aims to evaluate the epidemiological patterns, risk factors, microbiological profiles, and clinical characteristics of pediatric patients diagnosed with SA and OM. By analyzing demographic data, laboratory results, imaging findings, and treatment outcomes over 12 years, we seek to enhance our comprehension of the manifestation of these infections, their microbial etiologies, and the determinants affecting patient outcomes to enable early diagnosis and refine treatment techniques.

## Methods

### Study design and setting

This study employs a retrospective cross-sectional design to examine pediatric cases of SA and OM. The research was conducted at Bahrami Children’s Hospital, a teaching center affiliated with Tehran University of Medical Sciences (TUMS) in Tehran, Iran, from April 2011 to March 2023. The study protocol received approval from the Institutional Review Board and Ethics Committee of Tehran University of Medical Sciences, with a comprehensive informed consent process implemented for all participants (approval code: IR.TUMS.MEDICINE.REC.1401.657).

### Participants

Participants were carefully selected based on specific inclusion and exclusion criteria. Patients were included in the study if they met the following criteria: First, they must have had a discharge diagnosis of OM or SA, confirmed and coded according to ICD-10 classification (M86.1-M86.9 for OM; M00.0-M00.9 for SA). Second, patients needed to present with at least two clinical symptoms consistent with the diagnosis, including localized pain or tenderness, swelling and/or redness over the affected area, fever above 38.0 °C, limited range of motion or limping, or refusal to bear weight on the affected limb. Third, patients were required to have at least one positive diagnostic finding from the following categories:

1. Microbiological confirmation through either a positive culture from joint fluid, blood, or bone biopsy, or presence of bacteria on Gram stain of sterile fluid or tissue.
2. Characteristic joint fluid analysis showing purulent synovial fluid with WBC count exceeding 50,000 cells/µL with more than 90% polymorphonuclear cells.
3. Significant laboratory findings including elevated serum WBC count above 12,000 cells/µL, elevated CRP levels (qualitative values of 1 + or higher and quantitative values of 20 mg/L or higher), or ESR exceeding 40 mm/h.
4. Radiographic findings included either (a) ultrasonographic evidence of joint effusion, synovial thickening, or capsular distention suggestive of SA, or (b) MRI findings such as synovial enhancement (for SA) or increased signal intensity on T2-weighted sequences (for OM), as well as (c) plain radiographs demonstrating periosteal elevation, lytic lesions, or cortical bone destruction.

Ultrasound examinations were performed and interpreted by experienced pediatric radiologists following a standardized protocol adapted from Nguyen et al. [21]. High-frequency linear transducers (8–18 MHz) were used for most joints, while lower-frequency probes (6–15 MHz) were applied for deeper joints like the hip. Scans were obtained in standardized planes—anterior, posterior, and lateral views for the knee and ankle, and anterior and lateral views for the hip.

Minimal transducer pressure was applied to avoid fluid displacement, and dynamic, multi-focal scanning was performed, using the contralateral joint for comparison when needed. In hip evaluations, a capsular thickness ≥ 5 mm or a side-to-side difference > 2 mm was considered significant. For OM, sonographic findings such as soft tissue swelling, periosteal elevation > 2 mm, or subperiosteal fluid collections were suggestive of early infection and prompted further MRI evaluation when necessary.

Patients who had significantly incomplete medical records, previous history of open fracture, bone surgery, or nosocomial infection, or definitive diagnosis other than OM or SA excluded from the study. The Kocher criteria are utilized for acute SA of hip. Imaging is used as a supplementary diagnostic technique in unclear cases. Final diagnosis confirmed by a pediatric infectious disease specialist or pediatric rheumatologist.

### Data sources and measurement

Data were extracted from hospital records in the Bahrami Hospital database. A researcher-designed checklist was used to obtain the following variables: demographic data, medical history, clinical presentation, laboratory findings, radiographic evidences based on written reports by experienced radiologists, treatment details, and follow-up reports after discharge.

### Study size

The sample size was determined using the standard formula for prevalence studies, considering an expected prevalence (P) of 16% based on the study by Spyridakis et al. [2]. This calculation yielded a minimum required sample size of 52 patients. To account for potential dropouts and enhance statistical power, a total of 65 patients were included. Since the final sample size exceeded the minimum requirement, it was deemed sufficient to ensure the reliability and validity of the findings.

### Statistical analysis

Descriptive statistics were used to summarize patient demographic and clinical characteristics. Quantitative variables were reported as mean ± standard deviation (SD), and categorical variables were presented as frequency and percentage. To compare categorical variables, the Chi-square test or Fisher’s exact test was applied as appropriate. Continuous variables were analyzed using the independent t-test or one-way ANOVA for normally distributed data, and the Mann-Whitney U test or Kruskal-Wallis test for non-normally distributed data. A p-value < 0.05 was considered statistically significant. All statistical analyses were conducted using SPSS version 26 (IBM Corp., Armonk, NY, USA).

## Results

This retrospective study included 65 patients at Bahrami Hospital from 2011 to 2023, with 46 cases (70.8%) of SA and 19 cases (29.2%) of OM. None of the patients had concurrent septic arthritis and osteomyelitis.

The study population comprised 45 male patients (69.2%) and 20 female patients (30.8%). The mean age of patients with SA was greater than OM. Eight patients (12.3%) were hospitalized due to symptom recurrence. The mean duration of hospitalization for patients with SA was 8.13 days, whereas for patients with OM, it was 15.32 days (Table 1). The average duration of hospitalization for patients with comorbidities, shown in Table 1, was significantly longer than that for those without (18.4 days vs. 9.1 days; p: 0.004). Eighteen patients (27.7%) had recently used antibiotics following the onset of symptoms. Cefixime (38.9%), amoxicillin (16.7%), and penicillin (16.7%) were the most commonly used antibiotics.

**Table 1:** Demographic distribution of patients with SA and OM

Characteristic	Septic Arthritis (*n* = 46)	Osteomyelitis (*n* = 19)
Age (years)		
Mean ± SD	5.4 ± 4.3	4.8 ± 3.8
Newborn (0–28 days)	0 (0%)	0 (0%)
Infant (29 days − 1 year)	5 (10.9%)	4 (21.1%)
Toddler (1–3 years)	9 (19.6%)	7 (36.8%)
Preschooler (3–5 years)	16 (34.8%)	1 (5.3%)
School age (6–12 years)	16 (34.8%)	7 (36.8)
Gender, n (%)		
Male	33 (71.7%)	12 (63.2%)
Female	13 (28.3%)	7 (36.8%)
Residence, n (%)		
Urban	42 (91.3%)	18 (94.7%)
Rural	4 (8.7%)	1 (5.3%)
Infection type, n (%)		
Primary	42 (91.3%)	15 (78.9%)
Recurrent	4 (8.7%)	4 (21.1%)
Duration of hospitalization (days)		
Mean ± SD	8.1 ± 6.7	15.3 ± 10.6
Underlying Disease, n (%)		
Aplastic Anemia	0 (0%)	1 (2.2%)
Achondroplasia	1 (5.3%)	0 (0%)
Hepatitis B	0 (0%)	1 (2.2%)
Serum sickness	0 (0%)	1 (2.2%)
ALL	0 (0%)	1 (2.2%)
CP	0 (0%)	1 (2.2%)
Epilepsy	1 (5.3%)	0 (0%)
ESRD	0 (0%)	1 (2.2%)

Of the total patients, three cases (4.6%) had a history of immunosuppressive therapy. This included one patient with aplastic anemia under treatment with cyclosporine (50 mg/day) and prednisone (20 mg/day). The second case was a patient with a history of acute lymphoblastic leukemia (ALL) diagnosed 8 years prior, who was receiving mercaptopurine and methotrexate therapy. The third case had a history of serum sickness and was under treatment with methylprednisolone. In addition to these cases, two patients (3%) were receiving antiepileptic medications (phenytoin and phenobarbital).

In total, 10 patients (15.4%) reported a recent history of trauma to the affected limb (bone, joint). 24 patients (36.9%) reported experiencing a cluster of symptoms consistent with viral infections, including coryza, sore throat, and fever, within one month prior to the diagnosis of osteoarticular infection. The mean time from symptom onset to hospitalization for patients with SA was 7.1 days, and that for patients with OM was 9.8 days.

**Fig. 1:**
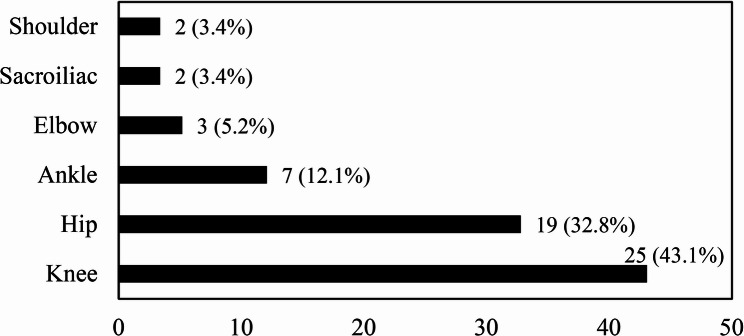
Distribution of joint involvement in patients with SA

**Fig. 2:**
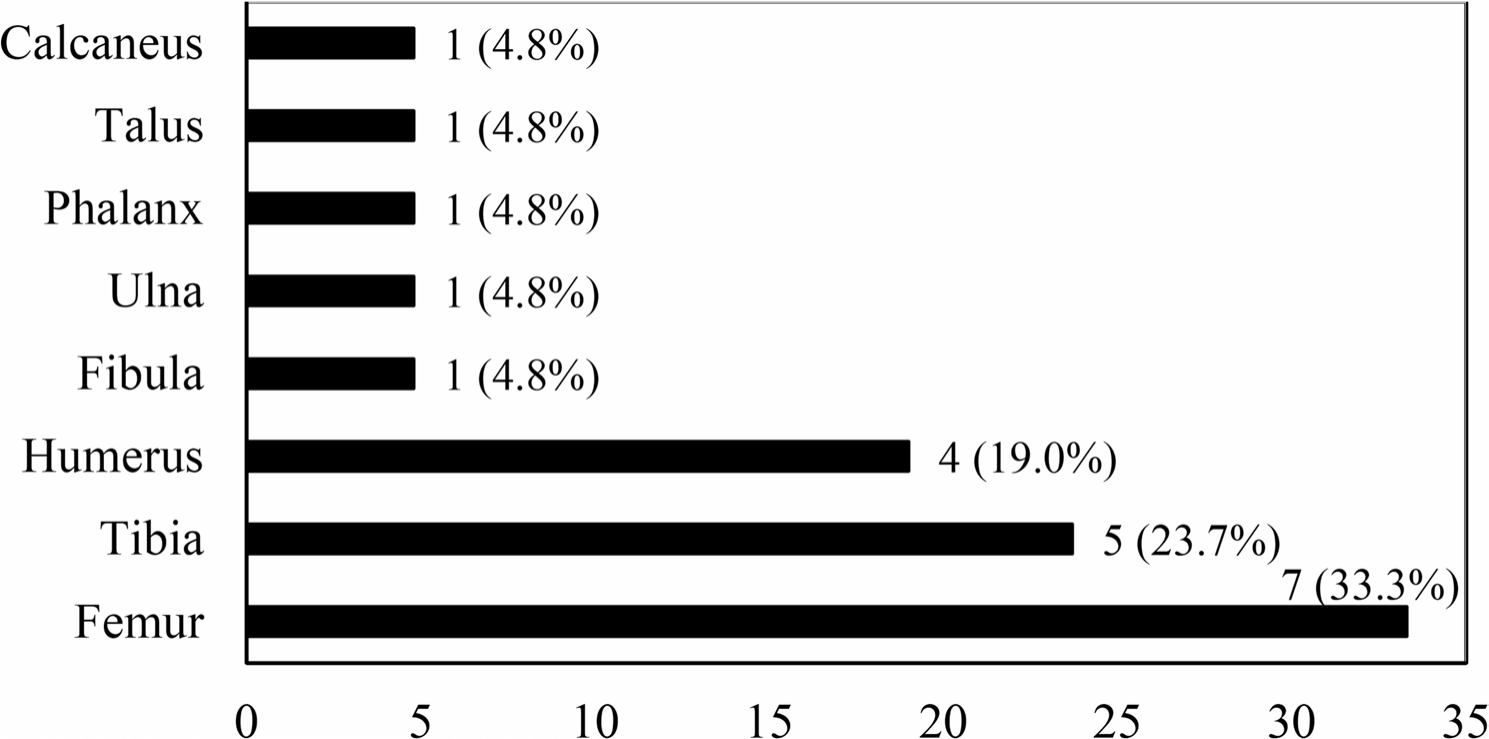
Distribution of bone involvement in patients with OM

73.9% of patients with SA had monoarticular involvement, whereas the remaining 26.1% had oligoarticular (two-joint) involvement. The most commonly affected joints were the knee (43.1%), hip (32.8%), and ankle (12.1%) (Fig. 1). Among the OM patients, except for two patients (10.5%) with simultaneous infection of two bones, the remaining patients (89.5%) had single bone involvement. The most frequently infected bones were the femur (33.3%), tibia (23.7%), and humerus (19%) (Fig. 2).

The most common clinical signs and symptoms in OM patients were pain or discomfort (100%), tenderness (89.5%), and fever (78.9%). For SA patients, the most common clinical signs and symptoms were pain or discomfort (100%), reduced range of motion (84.8%), and tenderness (78.3%) (Table 2). The mean temperature recorded upon admission was 38.0 °C (95% CI: 37.7–38.3) for patients with SA and 38.6 °C (95% CI: 38.2–39.0) for those with OM (Table 2).

**Table 2:** Frequency of clinical Signs, laboratory parameters in patients with OM and SA

Clinical Signs and Symptoms		Septic Arthritis (*n* = 46)	Osteomyelitis (*n* = 19)
Fever		29 (63.0%)	15 (78.9%)
Pain		46 (100.0%)	19 (100.0%)
Local signs		30 (65.2%)	13 (68.4%)
Tenderness		36 (78.3%)	17 (89.5%)
Decreased ROM^1^		39 (84.8%)	14 (73.7%)
Limping		32 (76.2%)	12 (80.0%)
Inability to bear weight		26 (61.9%)	6 (40.0%)
WBC (×10³ cells/µL)	Elevated (%)	50.0%	57.9%
	Mean ± SD	12.0 ± 5.5	13.5 ± 4.4
NLR^2^	Elevated (%)	63.0%	57.9%
	Mean ± SD	3.4 ± 3.1	2.9 ± 1.6
ESR^3^ (mm/h)	Elevated (%)	54.3%	52.6%
	Mean ± SD	46.3 ± 31.9	47.0 ± 31.1
CRP^4^ (mg/L)	Elevated (%)	80.4%	68.4%
	Mean ± SD	48.2 ± 34.8	50.9 ± 34.1
Hemoglobin (g/dL)		11.0 ± 1.3	11.0 ± 1.5
Platelet (×10³/µL)		353.9 ± 178.1	456.2 ± 192.8
Urea (mg/dL)		24.5 ± 20.8	22.3 ± 8.1
Creatinine (mg/dL)		0.6 ± 0.6	0.5 ± 0.1

Values are presented as Number (%) and Mean ± SD

^1^*ROM* range of motion

^2^*NLR* Neutrophil-to-Lymphocyte Ratio

^3^*ESR* Erythrocyte Sedimentation Rate

^4^*CRP* C-Reactive Protein

In OM patients, leukocytosis (WBC count > 12000) was present in 57.9% of cases, 52.6% had an ESR > 40 mm/h, and 68.4% had elevated CRP levels (qualitative values of 1 + or higher and quantitative values of 20 mg/L or higher).

Blood culture was positive in 23.1% of OM patients, and bone culture was positive in 20% of the patients whose culture reports were available in their hospital records. In patients with positive blood cultures, the most common identified organism was *Staphylococcus aureus* (75%). In OM patients with positive blood cultures, although the occurrence of clinical symptoms and increases in the WBC count, NLR, and inflammatory markers were more severe and frequent than those in culture-negative patients, statistical analysis revealed that only the increase in the ESR was significantly and nonrandomly associated with positive blood cultures (p: 0.045) (Table 2). In SA patients, leukocytosis was present in 50% of cases, 54.3% had an elevated ESR, and 80.4% had elevated CRP levels (Table 2). Among SA patients with available culture results, positive blood cultures and positive synovial fluid cultures were found in 13.3% and 23.5% of cases, respectively. In patients with positive synovial fluid cultures, *Staphylococcus aureus* (75%) was the most commonly identified organism. Similar to osteomyelitis patients, only the ESR showed a significant and nonrandom increase in association with positive cultures (p: 0.007) (Table 3).

**Table 3:** Distribution of clinical symptoms and laboratory parameters based on Blood/Synovial culture results

Clinical Signs and Symptoms		Osteomyelitis			Septic Arthritis	
	Positive Blood Culture (*n* = 4)	Negative Blood Culture (*n* = 9)	*p*-value	Positive Synovial Fluid Culture (*n* = 4)	Negative Synovial Fluid Culture (*n* = 13)	*p*-value
Fever	4 (100%)	7 (77.8%)	0.305	3 (75%)	12 (92.3%)	0.347
Local signs	3 (75%)	6 (66.7%)	0.764	4 (100%)	9 (69.2%)	0.205
Tenderness	4 (100%)	7 (77.8%)	0.305	4 (100%)	10 (76.9%)	0.305
Decreased ROM^1^	3 (75%)	6 (66.7%)	0.764	4 (100%)	12 (92.3%)	0.567
WBC (×10³ cells/µL)	12.9	11.7	0.445	14.5	11.2	0.211
NLR^2^	4.2	2.7	0.141	2.6	2.2	0.600
ESR^3^ (mm/h)	67.5	32.8	0.045	92.2	45.6	0.007
CRP^4^ (mg/L)	78.2	49.0	0.115	60.5	57.5	0.881

Values are presented as Number (%) and Mean ± SD

^1^*ROM* range of motion

^2^*NLR* Neutrophil-to-Lymphocyte Ratio

^3^*ESR* Erythrocyte Sedimentation Rate

^4^*CRP* C-Reactive Protein

To further explore the relationship between inflammatory markers and disease severity, the association between ESR and the duration of hospitalization was analyzed. Among patients with SA, those with elevated ESR had a significantly longer mean hospital stay compared to those with normal ESR (10.6 ± 8.0 days vs. 5.2 ± 3.1 days; p: 0.004). Similarly, in OM patients, elevated ESR was associated with a significantly longer hospitalization period (18.1 ± 12.9 days vs. 12.2 ± 6.6 days; p: 0.033).

In the ultrasound examination of 35 patients with SA, 26 patients (74.3%) had joint effusion, 10 patients (28.6%) had increased synovial thickness or joint capsule swelling, and 8 patients (22.8%) had normal ultrasound findings or no findings suggestive of SA. Among the 46 patients with SA included in this study, 18 patients underwent plain radiography of the affected limb, with 11 patients (61.1%) reported as normal and 7 patients (38.9%) showing soft tissue swelling accompanied by decreased bone density. The mean hospitalization duration for SA patients with abnormal ultrasound or X-ray findings was longer than that for those with normal imaging results (9.3 days vs. 3.8 days; p: 0.005). The most common findings in plain radiographs of 11 OM patients were the formation of osteolytic foci (54.5%) and soft tissue edema (36.4%). Three patients (27.3%) had normal radiographs. MRI reports of 8 OM patients revealed increased signal intensity in the affected bone, the most important diagnostic finding, in 7 patients (87.5%). Periosteal reactions (50%) and the presence of abscesses or collections (37.5%) were other reported findings in these patients. According to hospital records, 11 out of 19 OM patients underwent bone scans, all of which (100%) reported increased uptake of contrast material by the affected bone as a diagnostic finding. The most common antibiotic regimens used for patients were ceftriaxone + cloxacillin (27.3%), cloxacillin (22.7%), and ceftriaxone + clindamycin (15.9%) (Table 4).

**Table 4:** Antibiotic regimens used in patient treatment

Antibiotic Treatment	Number (%)
Ceftriaxone + Cloxacillin	12 (27.3%)
Cloxacillin	10 (22.7%)
Ceftriaxone + Clindamycin	7 (15.9%)
Ceftazidime + Vancomycin	3 (6.8%)
Ceftriaxone + Cefotaxime + Clindamycin	2 (4.5%)
Ceftriaxone + Vancomycin	2 (4.5%)
Clindamycin	2 (4.5%)
Cefotaxime + Clindamycin	1 (2.3%)
Cefotaxime + Cloxacillin	1 (2.3%)
Cefotaxime	1 (2.3%)
Ceftriaxone + Cloxacillin + Vancomycin	1 (2.3%)
Cloxacillin + Amikacin	1 (2.3%)
Meropenem + Linezolide	1 (2.3%)

## Discussion

The present study aimed to investigate the demographic, clinical, laboratory, and imaging characteristics of patients with SA and OM. The findings reveal significant similarities and discrepancies with prior research. The prevalence of SA (70.8%) exceeded that of OM (29.2%), which aligns with the findings of previous studies indicating that SA is more prevalent in children. However, some studies have reported that the prevalence of OM is similar to or even greater than that of SA [9, 10, 17, 22]. These discrepancies may stem from variations in diagnostic criteria, study populations, and access to healthcare services. Both diseases were more prevalent in male children, which is consistent with the epidemiological patterns reported in most studies. This sex difference may result from differences in physical activity, exposure to trauma, or hormonal factors, which warrant further investigation [1, 4, 6, 8]. The mean age was greater in patients with SA than OM. Similar to several studies, the 2–14 year age group was the most common age group for patients [6, 11, 17]. The mean time from symptom onset to hospital admission was high in both groups. According to previous studies, treatment outcomes are significantly better in patients who present within one week of symptom onset than in those who present later. Our findings suggest that the presence of specified comorbidities significantly prolonged the duration of hospitalization (18.4 days vs. 9.1 days; *p* = 0.004). The presence of underlying diseases and use of immunosuppressive medications may pose diagnostic challenges in osteoarticular infections, potentially leading to delayed initiation of appropriate treatment and consequently extended hospital stays. A history of trauma (15.4%) and symptoms similar to viral respiratory or gastrointestinal infections prior to limb involvement (36.9%) highlight the importance of obtaining a detailed medical history and considering OM and SA as two important differential diagnoses in these patients [1, 6]. In our study population, no cases of concurrent SA and OM were identified. This could be attributed to our relatively small sample size. According to previous studies, concurrent osteoarticular infections are associated with more severe clinical manifestations and poorer outcomes [23, 24]. In this study, similar to previous studies, the knee, hip, and ankle were the joints most commonly affected in patients with SA, whereas the femur, tibia, and humerus were the bones most commonly affected in patients with OM. This similarity in the distribution pattern of joint and bone involvement in patients from this region with the global pattern is noteworthy [1, 8, 10, 14]. Although pain was the most common symptom in both diseases, other symptoms exhibited different patterns [8]. In patients with OM, tenderness and fever were more common, whereas in patients with SA, a reduced range of motion was more prominent. These differences can assist physicians in the initial and differential diagnosis of these two diseases. Fever was observed in fewer than 80% of the patients with both diseases, which contrasts with the findings of several previous studies reporting fever as the most common symptom or present in more than 80% of patients [1, 4]. This difference may be attributed to prior antibiotic use (27.7%) or delayed presentation. Elevated WBC counts and inflammatory marker levels showed different patterns in both diseases. In patients with OM, the mean of WBC count was higher compared to those with SA [1, 13]. In this study, two laboratory parameters, ESR and CRP, were analyzed across different patient groups. In both diseases, a higher percentage of patients exhibited elevated CRP compared to elevated ESR. This finding aligns with the 2023 guideline for diagnosis and management of acute bacterial arthritis by the Pediatric Infectious Diseases Society (PIDS) and the Infectious Diseases Society of America (IDSA), which recommends measuring serum CRP levels in suspected cases as a baseline finding for subsequent monitoring and decision-making, despite its noted low diagnostic accuracy [25]. However, in our comparative analysis between patients with positive blood or synovial fluid cultures and those with negative cultures, only ESR levels showed a statistically significant difference between groups (*p* < 0.05). A study conducted in Singapore reported a higher incidence of sequelae in SA patients with positive fluid cultures compared to those with negative cultures [4]. Furthermore, Chen et al. found that culture-positive patients had significantly higher rates of surgical intervention and complications than their culture-negative counterparts [15]. These findings underscore the clinical utility of ESR as a surrogate marker for infection severity, particularly when cultures are negative or delayed. Moreover, our study revealed a statistically significant positive correlation between elevated ESR levels and longer hospital stay, suggesting that ESR may serve not only as a diagnostic marker but also as a potential prognostic indicator of disease severity. This could assist clinicians in early risk stratification, and in planning both the duration and intensity of treatment and follow-up care.

Further studies with larger sample sizes are recommended to validate these findings. *Staphylococcus aureus* was the most common pathogen in both diseases, which is consistent with the global pattern and emphasizes the importance of appropriate antibiotic coverage for this pathogen [2, 3, 7, 10, 14, 22]. The positive culture rate in this study was relatively low, similar to some comparable studies but contrary to other studies [7, 14]. This finding underscores the importance of prompt empirical treatment initiation, even in the absence of positive culture results. This low rate could be due to prior antibiotic use or delayed sampling. However, in the study by Lansell et al., no significant associations were found between blood, bone, and joint fluid culture results in patients with OM and SA and prior antibiotic use [11]. Statistical analysis indicated that the presence of involvement in ultrasound or X-ray findings is directly related to the severity and activity of the disease, which can guide treatment plans. Conversely, although the detection rate of OM via X-ray findings of the affected limb was higher than that reported in Fan’s study, both studies indicated that this modality lacks sufficient sensitivity in diagnosing OM [19]. In patients with OM, MRI demonstrated high sensitivity which is consistent with previous studies that introduced MRI as the gold standard for diagnosing OM [3, 6, 9, 13]. All patients received intravenous antibiotic treatment during their hospital stay. The combination of ceftriaxone and cloxacillin (26.1%) was the most common antibiotic regimen used in this hospital, providing adequate coverage for common pathogens, especially *Staphylococcus aureus*. However, there is a need for periodic reviews of antibiotic resistance patterns in each region. The results of several clinical trials have shown that in patients with uncomplicated OM and SA, a short course of intravenous antibiotics followed by oral antibiotics is safe and effective. Evaluating the effectiveness of this treatment protocol in future studies is recommended [16]. In our study protocol, all patients presenting with fever, pain, point tenderness, weight-bearing intolerance, and leukocytosis were initially managed as acute bacterial osteomyelitis to prevent potential complications. Among these patients, two cases were particularly noteworthy. Initially treated with antibiotics for suspected acute hematogenous osteomyelitis (AHO), they showed temporary clinical improvement. However, they later experienced symptom recurrence with multifocal involvement. Further evaluation, including negative bone cultures and biopsies ruling out infection and neoplasm, led to the diagnosis of chronic recurrent multifocal osteomyelitis (CRMO), also known as chronic nonbacterial osteomyelitis (CNO). CRMO is characterized by subacute or chronic inflammation with gradual onset of pain, swelling, and tenderness in bones, usually symmetrically and multifocally, particularly in the clavicle. The diagnosis is made through a bone biopsy, ruling out infectious and neoplastic issues [20, 26]. This highlights that while early empiric antibiotic therapy is crucial in suspected osteoarticular infections, persistent or recurrent symptoms, especially with multifocal involvement and negative cultures, should prompt consideration of non-infectious etiologies such as CNO. Both patients were successfully treated with methotrexate and remained symptom-free after treatment discontinuation two years prior.

This study has several limitations. Its retrospective nature may have led to the loss of some data. Additionally, conducting the study in a single medical center may limit the generalizability of the results. For future studies, it is suggested that antibiotic resistance patterns, long-term treatment outcomes, disease complications, and particularly cases with concurrent SA and OM be investigated to better characterize their clinical features and risk factors. In conclusion, this study demonstrated that SA and OM, despite their similarities, have significant differences in terms of clinical manifestations and paraclinical findings. Attention to these differences can contribute to faster and more accurate diagnoses and, consequently, more effective treatment of these diseases. Furthermore, the association of elevated ESR levels with both prolonged hospital stay and positive blood or synovial fluid cultures—as indicators of disease severity—highlights the critical role of this marker in patient management and clinical decision-making.

## Data Availability

No datasets were generated or analysed during the current study.

## Abbreviations

SA: Septic Arthritis
OM: Osteomyelitis
AHO: Acute Hematogenous Osteomyelitis
ESR: Erythrocyte Sedimentation Rate
CRP: C-Reactive Protein
NLR: Neutrophil-to-Lymphocyte Ratio
ROM: Range of Motion
MRI: Magnetic Resonance Imaging
MRSA: Methicillin-Resistant Staphylococcus Aureus
CRMO: Chronic Recurrent Multifocal Osteomyelitis
